# The Dome: a virtual reality apparatus for freely locomoting rodents

**DOI:** 10.1016/j.jneumeth.2021.109336

**Published:** 2021-08-26

**Authors:** Manu S. Madhav, Ravikrishnan P. Jayakumar, Shahin G. Lashkari, Francesco Savelli, Hugh T. Blair, James J. Knierim, Noah J. Cowan

**Affiliations:** 1 Mind/Brain Institute, Johns Hopkins University, Baltimore, MD, U.S.A.; 2 Kavli Neuroscience Discovery Institute, Johns Hopkins University, Baltimore, MD, U.S.A.; 3 Laboratory for Computational Sensing and Robotics, Johns Hopkins University, Baltimore, MD, U.S.A.; 4 School of Biomedical Engineering, Djawad Mowafaghian Centre for Brain Health, University of British Columbia, BC, Canada; 5 Mechanical Engineering Department, Johns Hopkins University, Baltimore, MD, U.S.A.; 6 Department of Psychology, University of California Los Angeles, Los Angeles, CA, U.S.A.; 7 Francesco Savelli is currently affiliated with the Department of Biology, The University of Texas at San Antonio, San Antonio, TX, U.S.A.

**Keywords:** Virtual reality, cognitive map, control theory, multisensory integration

## Abstract

The cognitive map in the hippocampal formation of rodents and other mammals integrates multiple classes of sensory and motor information into a coherent representation of space. Here, we describe the Dome, a virtual reality apparatus for freely locomoting rats, designed to examine the relative contributions of various spatial inputs to an animal’s spatial representation. The Dome was designed to preserve the range of spatial inputs typically available to an animal in free, untethered locomotion while providing the ability to perturb specific sensory cues. We present the design rationale and corresponding specifications of the Dome, along with a variety of engineering and biological analyses to validate the efficacy of the Dome as an experimental tool to examine the interaction between visual information and path integration in place cells in rodents.

## 1. Introduction

While the Holodeck technology of Star Trek lore remains elusive, current virtual reality (VR) technology can nevertheless immerse our senses in simulated worlds that can be surprisingly convincing. To an experimental biologist, VR provides the facility to simulate crucial aspects of an animal’s natural environment, affording control of the information available to an organism regarding its state relative to the simulated world. The ability to seamlessly create conflicts between sensory streams while measuring behavioral output and internal neural representations allows researchers to understand the behavioral and neural mechanisms for integrating and re-weighting this sensory information [1, 2, 3]. The ability to manipulate and perturb these sensory inputs, in a manner that ensures the organism does not experience jarring discord between the senses, presents an opportunity to understand how environmental feedback is incorporated into neural computation.

VR has been applied to great effect in recent years as a powerful experimental tool [4, 5, 6, 7, 8, 9, 10, 11] to investigate neural and behavioral correlates of the ‘cognitive map’ in rodents [12]. According to O’Keefe and Nadel, elements of the cognitive map form the foundation of memory formation and retrieval [13, 14, 15], and spatial navigation [16]. During such navigation, neural correlates of the cognitive map can be found in the firing activity and local field potentials in the hippocampal formation. These correlates indicate the presence of an abstract, map-like representation of the external world. Place cells of the hippocampus in particular exemplify this abstract representation. The firing of these cells can be modulated by a variety of external inputs—e.g. landmarks, borders, direction, illumination, color, smell, taste, speed, distance, route—but does not require any one of these inputs for the stability of spatial tuning.

Rodent VR typically involves rats or mice locomoting on treadmills or air-cushioned balls; in such systems, the animal remains stationary in physical space while a visual display surrounding the animal is controlled by the motion of the treadmill or ball (for a review and some important exceptions, see [4, 17, 11]). This form of VR offers many advantages, such as the ability to present an infinite, scalable world in a manner that allows the use of bulky imaging equipment (e.g. two-photon imaging) that require a head-fixed preparation [18, 19, 20]. However, these head-fixed and body-fixed systems limit 35 locomotor performance and deprive the animal of the full range of sensory cues typically available during locomotion. For example, vestibular and other inertial inputs may be constrained and movements can be unnaturalistic. Why is this important? The cognitive map is a highly nonlinear dynamical system involving an intricate network of closed loops between myriad brain regions and sensory feedback. An experiment that enforces unnatural constraints can alter the operating point of this network in unintended ways. Such constrained experiments can generate important new understanding about a system, but given the existence of complex, nested feedback hierarchies and emergent non-linearities, the actual response of a system may differ in unexpected ways from predictions generated from highly constrained preparations [21]. Thus, complementary experiments that maintain the naturalistic feedback topology are essential to understanding multimodal interactions [22]. The design of the Dome apparatus was thus guided by the belief that—while recognizing the power and utility of head-fixed experiments—experiments that maintain naturalistic locomotion including intact path integration inputs are essential to understand the dynamics of cognitive representations in an ethological context [23]. To this end, the Dome is a VR apparatus that preserves naturalistic locomotion while presenting sensory conflict to a running rat. Within this apparatus, multiple path integration modalities can be manipulated and, if desired, placed in conflict with one another. In this manuscript, we report the hardware and software specifications as well as the design philosophy behind the Dome.

## 2. Design overview and subsystems

As with all experimental systems, the Dome has undergone many iterations, upgrades, and improvements. We focus in this paper on our most recent system, referred to as the *Current* version. However, as needed, we also describe a previous design iteration, referred to as the *Legacy* version. The *Legacy* design was used in our previous manuscript [10] and is the source of the neural validation data. The CAD plans, bill of materials, experimental control software and analysis software for the Dome are available under a Creative Commons Attribution-ShareAlike (CC BY-SA 4.0) license.

**Figure 1:**
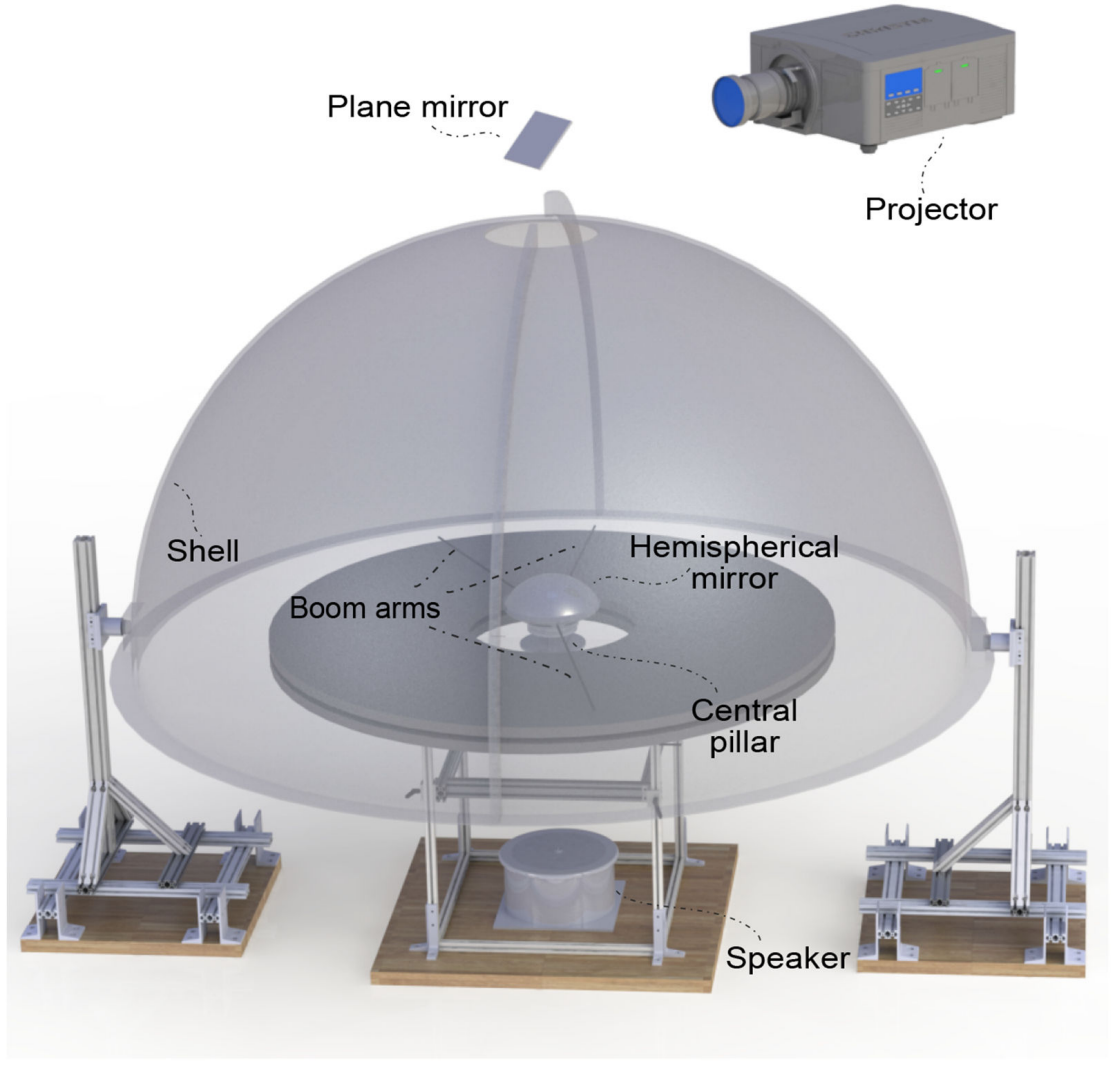
Rendered view of the dome apparatus. Note that the dome shell is opaque but was rendered semi-transparent for illustration. The projector (top) produces an image that gets reflected by an angled plane mirror, passes through the hole at the top of the shell, and onto a hemispherical mirror. The image is thus projected onto the inside surface of the shell. The shell is supported on two rigid support legs. The table inside the dome has a similar support structure. Three boom arms are attached to the central pillar which also supports the spherical mirror. Two of these arms are in front of and behind the rat respectively. The third arm serves to clean the table during experiments.

The Dome is a planetarium-like apparatus (Fig. 1) named for its most visible primary component, a large fiberglass hemispherical shell. This shell is mounted on two support legs. A projector mounted above the shell projects an image through a circular hole at the top of the shell; this image strikes a hemispherical mirror located at the center of curvature of the shell. The spherical mirror reflects the projected image onto the inside surface of the Dome, providing controllable visual cues 360° in azimuth and almost 90° in elevation. To allow for electrical commutation, the spherical mirror is mounted atop a rotating central pillar that passes through a hole in the middle of a circular table. A set of radial boom arms project outwards from the central pillar to the edge of the table. The surface of the table defines the laboratory *x*–*y* plane and the axis connecting the center of the shell and the hole on top of the shell defines the laboratory *z* axis. The rat runs on the table in a circular trajectory near the edge of the table. The control software, based on the Robot Operating System (ROS) framework, is modular, extensible, and open-source. The modular nature ensures that components of the software (called nodes) can be readily deployed on another apparatus that shares our design fully or partly. Nodes can also be added into the framework to enable new manipulation modalities and sensors. The software is also traceable and debuggable, with all communication between modules timestamped and logged during experiments.

### 2.1. Projection Shell

**Figure 2:**
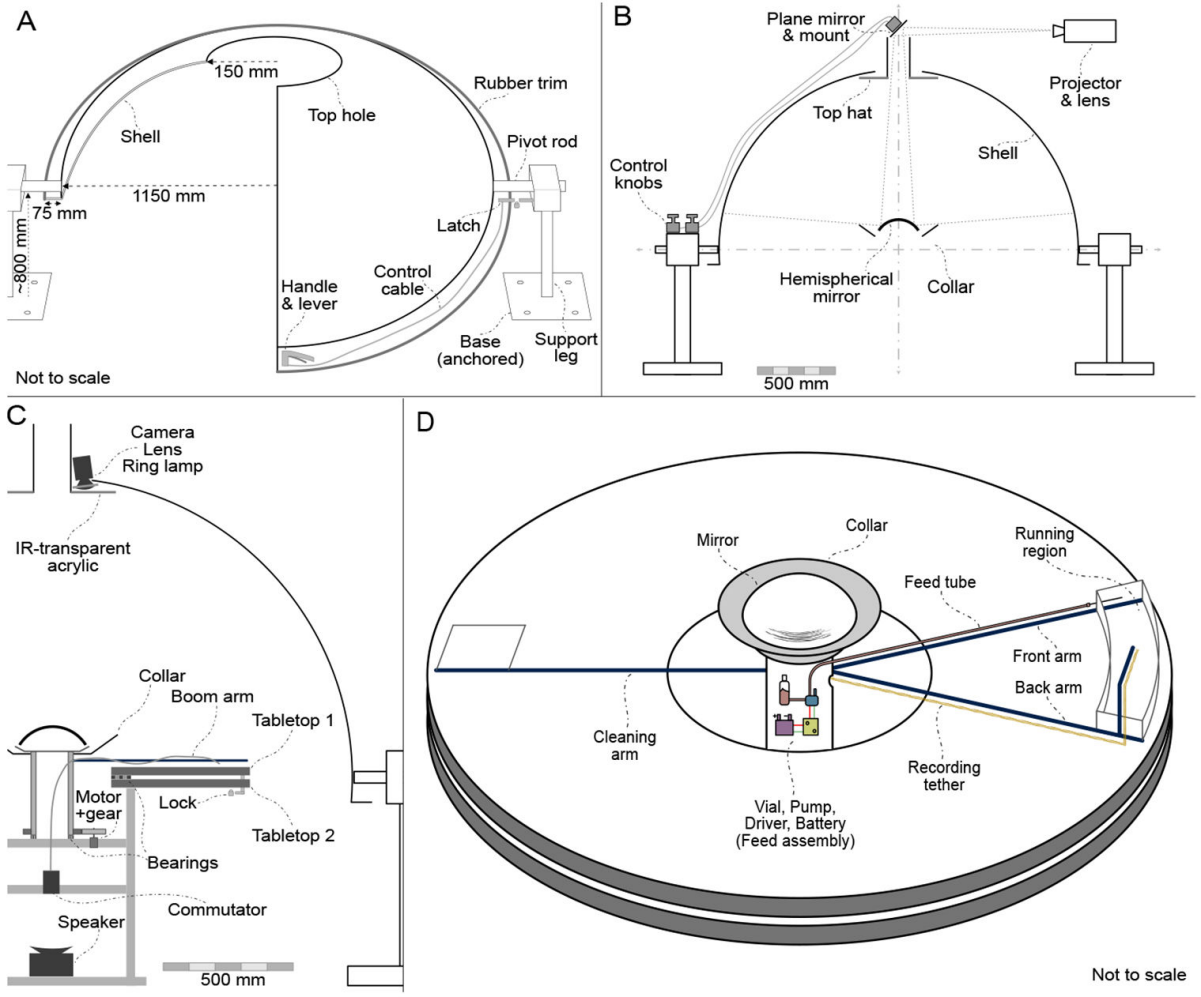
Dome subsystems. (A) Dome shell and support legs. Note that a sectioned view of the shell is shown. The support legs are anchored to the floor of the laboratory, and the shell can pivot on the support legs. The handle, control cable and latch are used to lock or pivot the Dome. (B) Projection system. The plane mirror can be tilted in two axes using control knobs; this can be used to adjust image alignment. The top hat blocks the view of the rat above the Dome, and the collar blocks its view of the spherical mirror. Figure is to scale. (C) Side view. A camera is mounted above the top hat to view and track the rat. The central pillar is rotated using a motor and gear system. Control signals (e.g. for feeding) can be sent to, and neural signals can be received from, the rotating central pillar through a commutator placed beneath the central pillar. A white-noise producing speaker is placed at the bottom center of the apparatus. Figure is to scale. (D) Table top. The rats runs in the enclosed region between the front and back boom arms. The recording tether is laid out along the back boom arm, whereas the feed tube is routed along the front arm. The feed assembly is mounted on the rotating central pillar.

The custom hemispherical shell (Immersive Display Group, Essex, UK) is made of fiberglass and is 2.3 m in diameter. The shell has elevation slightly more than 90°. The shell is manufactured in two halves for shipping and must be assembled on site. The seams are patched (using Bondo Fiberglass Resin Repair, 3M Inc., MN, USA) and sanded to match the curvature of the inner surface. The interior hemispherical surface is painted with a 50% reflective projector screen paint (RAL7040), as recommended by the manufacturer. Two short circular shafts embedded in the fiberglass at diametrically opposite points (Fig. 2A) mount the shell to circular bearings on the Dome support legs. This allows the Dome to be pivoted up for ease of access and pivoted down to its normal horizontal configuration used in experiments. The support legs, constructed from extruded aluminum components (80/20 Inc, IN, USA), are bolted to the floor of the laboratory. A fiberglass flange extends about 10 cm outward from the bottom of the shell. A latch (#2206A23, McMaster Carr, IL, USA) between a rigid support point (e.g. a wall or a floor mounted support post) and the flange locks the shell in a consistently repeatable horizontal configuration. A lever handle (#3790K11, McMaster Carr, IL USA) attached to the front of the flange operates the latch, allowing a single operator to raise and lower the Dome. Gas springs (#4155T901, McMaster Carr, IL, USA) connect the flange to the support legs, providing smooth and slow raising and lowering. Rubber trim (#8507K21, McMaster Carr, IL, USA) attached to the flange of the Dome mitigates the consequences of accidental bumps. The projector beam enters the Dome through a circular hole (30 cm dia.) on top of the Dome as mentioned above. Overhead cameras mounted to the periphery of the hole view and track the behavior of the animal.

### 2.2. Projection system

A projector (G7500UNL, Epson Inc., Nagano Japan), fitted with a long-throw lens (ELPLM11, Epson Inc., Nagano Japan), is mounted above the Dome. The projector sits horizontally on a shelf (wall- or ceiling-mounted), and a first-surface plane mirror (152 mm × 152 mm × 12.7 mm, First Surface Mirror LLC, OH, USA), mounted at an angle of approximately 45° directly above the dome, reflects the image down into the top hole of the shell. The angle of the plane mirror can be fine-tuned in two axes using an adjustable mirror mount (625-RC4, Newport Corp., CA, USA) and two remote control cables (RC-10, Newport Corp., CA, USA). If space and focal distance allows, the projector can be mounted vertically along the *z* axis, avoiding the need for the plane mirror. A hemispherical first-surface mirror (254 mm dia., 150 mm radius of curvature, 40/20 surface quality, 1/4-wave accuracy, protected aluminum coating, Cumberland Optical, MD, USA) is mounted at the center of the Dome, approximately at the height of the running surface; this mirror reflects the image onto the interior surface of the dome. The projector lens was chosen such that the size of the image formed on the hemispherical mirror is less than its diameter (approximately 203 mm image height), and the resulting image can be focused on the inside surface of the shell. Two other custom components aid with the optics. A ‘top hat’ mounted to the shell hole has opaque cylindrical sides tall enough to block the line of sight of the rat to any stationary laboratory cues that may be visible through the top hole. An opaque ‘collar’ placed around the hemispherical mirror blocks everything but the central circular part of the projector’s rectangular image and also prevents bright reflections from the hemispherical mirror from directly impinging on the rat’s eye (Fig. 2B).

### 2.3. Table

Two circular wooden tabletops (150 cm dia., 38 mm thick) with a central hole (45 cm dia.) are connected axially through a ring bearing or ‘lazy susan’ (80 cm dia., 12.7 mm thick, VXB Ball Bearings, CA, USA) (Fig. 2C,D). The bearing allows the upper tabletop to rotate on top of the fixed lower tabletop. The tabletops can be locked together by the experimenter at 8 equally spaced angular displacements. This feature allows the table orientation to be fixed in any experimental session but randomized between experimental sessions to control for any local olfactory or textural cues. The tabletop can also be motorized to enable manipulation of physical cues (e.g. vestibular) during the experiment, although this feature has yet to be added. A black laminate covering on the tabletops makes the floor texture uniform throughout, minimizes local optic flow, and enables easy cleanup and sanitization. The lower tabletop is rigidly attached to a central support structure (80/20 Inc.) which is in turn anchored to the floor of the laboratory (Fig. 2C). A centrally mounted speaker below the table provides white noise during the experiments, but can also be used to provide non-directional auditory cues (Fig. 2C). A ring of speakers can be readily mounted below the bottom table if directional audio cues are required (e.g. to provide auditory landmarks). Curtains hang below the rim of the table inner hole and from the Dome flange to the ground; these help prevent the rat from seeing cues in the center of the table and the support legs. A thick curtain hangs around the Dome outer shell and provides both light and sound isolation.

### 2.4. Central pillar

The central pillar is a rotating hollow metal cylinder that also serves as the mounting location for the hemispherical mirror(Fig. 2C,D). Below the mirror, a set of radial boom arms extend from the central pillar towards the edge of the table. A pump, controller, vial of liquid feed and battery are mounted on the central pillar, along with any other accessories that need to rotate along with the rat. The central pillar rotates on a ring bearing on the central support structure. When wired recording is used, neural recording tethers can be passed through the hollow portion of the central pillar and attached to an electrical commutator (PSR-36, Neuralynx, MT, USA). In a wireless configuration, a smaller commutator can be substituted to pass on any control signals to the rotating stage(e.g. for feeding). A high-torque motor (DCX22S, Maxon, Friedrichsdorf, Germany) and gearhead(GPX22, Maxon, Friedrichsdorf, Germany) with a built-in optical encoder (ENC 30 HEDL 5540, Maxon, Friedrichsdorf, Germany) are used to rotate and determine the angle of the central pillar. A magnet on the central pillar passes by a hall-effect sensor (55100-3H-02-D, Littelfuse, IL, USA) every rotation, which can be used by the motor controller as an angular zero calibration for the laboratory reference frame. The metal support legs as well as the central support structure are grounded in order to prevent interference in neural recordings.

In the *Legacy* version, the rotating neural commutator itself was used as the central pillar. We adopted the current design since it allows us to swap in a different commutator or neural recording system, and avoids heavy axial loads on the commutator bearings which they are typically not designed to withstand.

### 2.5. Boom arms and feeding

The current experimental configuration uses three radial boom arms: the front arm, the back arm, and the cleaning arm that are mounted in front of, behind, and across from the rat, respectively (Fig. 2D). The boom arms extend from the central pillar to the edge of the tabletop (Fig. 2C). Carbon fiber rods (6 mm dia., Goodwinds Composites, WA, USA), serve as radial boom arms. The arms are supported towards the middle of the table by rubber wheels to prevent sagging and are equipped with mating connectors and extensions to facilitate attachment of other components.

Two transparent polycarbonate walls that are mounted between the front and back arms restrict the radial movement of the rat. However, the rat is free to run in the circular track. As the rat runs, the central pillar rotates and the boom arms move along with the animal.

The front arm is used to route a feeding tube and the back arm is used for routing cables for wired neural recordings. An additional component attached to and cantilevered above the back arm guides a neural recording tether and suspends the recording headstage above the rat (see below for details about neural recording hardware). A micro peristaltic pump (RP-Q1, Taskago Fluidics, Aichi Japan), pump driver, battery, and feed vial are mounted on the central pillar. Liquid feed (50% diluted Ensure®) is pumped from the vial into a feed tube that is routed along the front boom arm to a feeding needle (FTSS-16S-76, Instech Labs, PA, USA) that drops the feed onto the table in front of the running rat (Fig. 2D). A sweeper, composed of a plastic spreader and paper towels, is attached to the cleaning arm. It wipes up or spreads out the scent of urine and uneaten food, as well as pushes feces off the table. The sweeper reduces the salience and stability of local olfactory cues that may provide uncontrolled spatial information to the animal.

### 2.6. Camera and tracking

A high-resolution (2048 px × 2048 px), high-frame rate (45–90 fps) NIR camera (GS3-U3-41C6NIR-C, FLIR, OR, USA) with a wide-field lens (NMV-6M1, Navitar, NY, USA) records the animal behavior. The camera is mounted to the outer periphery of the top hole in the Dome shell. Since the camera is not mounted in the center of the hole, it can be a polarizing visual cue. In order to prevent this, we hide the camera from the rat by placing it behind the ‘brim’ of the top hat. In the *Current* version, we track the head position of the rat in real-time using the camera [24]. To enable this, the neural recording headstage attached to the head of the rat supports a lightweight three-dimensional pattern of retro-reflective markers (the ‘crown’). A ring of NIR LEDs (QBLP670-IR3, QT Brightek, CA, USA) placed around the camera lens serves as the IR light source. The top hat brim is made of an IR transmitting acrylic sheet which is opaque to visible light (#3143, ePlastics, CA, USA); thus the rat cannot see the camera. The infrared LED light readily passes through the acrylic sheet into the interior of the Dome, illuminates the retro-reflective markers, and passes back through the acrylic to the camera (Fig. 2C). A methodological description of the crown tracking system will be published as a separate article.

### 2.7. Neural recording setup

**Figure 3:**
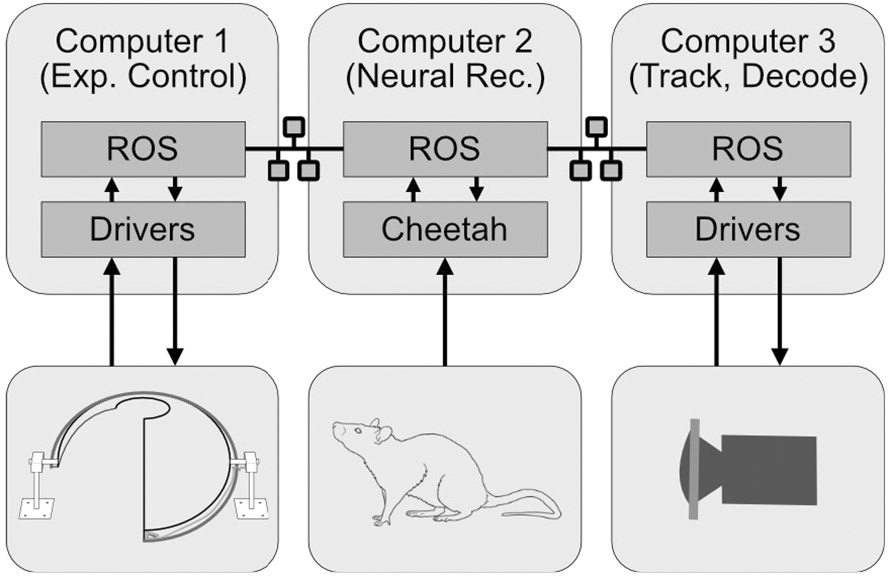
Software Architecture. Three networked computers are used to run the Dome experiments. They communicate through the ROS framework, which then interfaces with lower-level software. Computer 1 is primarily used for experimental control and generating the projected visual image. Computer 2 performs the neural signal acquisition recording. Computer 3 is used for processing and saving camera frames, tracking rat position, and for neural decoding when applicable.

A wired system (Digital Lynx SX, Neuralynx, MT, USA) is used for neural recordings. The commutator placed below the central pillar rotates to avoid twisting the recording tether. The Dome can also be operated in a semi-wireless recording mode using a wireless transmitter (FreeLynx, Neuralynx, MT, USA). In this mode, the wireless transmitter and battery are placed on the central pillar, eliminating the need for a high-channel-count neural commutator. Wireless data loggers and head-mounted wireless transmitters can also be used—this completely avoids the rat being tethered to the back boom arm. The hollow but rotating design of the central pillar allows flexibility in the choice of tethered or wireless recording solutions. The recording system is controlled by the Neuralynx Cheetah software running on a computer with the Windows 10 operating system (Computer 2, Fig. 3).

### 2.8. Software and Control

Two computers running Ubuntu Linux (16.04 Xenial Xerus) are used for primary experiment control (Computer 1, Fig. 3) and video tracking of rat position and head direction (Computer 3, Fig. 3). The primary framework of experiment control is Robot Operating System (ROS) [25, 26]. Originally developed and largely used to operate robotic platforms, ROS offers a powerful cross-platform interface for several independently running programs (nodes) on a set of networked computers to communicate with each other by passing information (messages) on communication channels (topics). The nodes can be written in a variety of languages (C++, Python, MATLAB). The fact that each node operates independently of others makes it extremely flexible in terms of modifying the experiment control, adding and modifying software features, and incorporating new sensors and actuators. ROS is thus well suited for experimental control, especially in a networked multi-computer multi-operating system scenario such as ours. ROS can also operate with various real time linux kernels that enables us to perform fixed-latency data acquisition.

Computer 1 uses a data acquisition system (DAQ PCIe-6259, National Instruments, TX, USA) to communicate with the Dome apparatus, interfaced through a real-time linux kernel (Xenomai v3.1 patched on Kernel v4.19.66) and ROS. The optical encoder and hall-effect sensor signals are inputs to the DAQ, and the feeding pump and central pillar motor signals are outputs from the DAQ. The projector is configured as one of the displays of Computer 1, and the visual scene was generated using OpenGL running within a ROS node. Arbitrary shapes can be displayed through OpenGL at high frame rates by the use of pre-compiled display lists. Computer 3 (Ubuntu 18.04) receives the camera signal through a USB3.0 interface and runs a custom-developed ROS node that tracks the position and orientation of the animal’s head [24]. In addition, it also records high-resolution video at full frame rate (45–90 fps) for post-experiment processing.

### 2.9. Synchronization and Data acquisition

Experimental function and data integrity demands that the device clocks on all three computers be kept precisely synchronized. This is accomplished in multiple stages. The Network Time Protocol (NTP) is used by the operating systems to synchronize their respective system clocks to Computer 1, which acts as the local NTP server. In addition, a randomized TTL pulse train (mean 10 s. between pulses, 1 s. pulse duration) generated from Computer 1 is fed into the digital inputs of the neural acquisition system in Computer 2. The paired timestamps of these pulses are then used post-hoc to synchronize the neural and experimental data streams using the Needleman-Wunsch algorithm [27]. In addition, the hall-effect sensor is fed into a separate digital input on the neural recording system as an independent synchronization backup that generates one TTL pulse every lap.

Data is acquired on all three computers independently. Computer 1 and Computer 3 record and timestamp all the data flowing between their ROS nodes in the ROSbag format. This includes all communication between nodes, inputs to and outputs from the DAQ on Computer 1, and the camera frames on Computer 3. Computer 2 records neural data in Neuralynx custom formats, including the timing of the digital synchronization signals. Since the experimental and neural recording systems are largely independent, the neural recording system can be swapped out for any other system that can accurately timestamp TTL pulses.

### 2.10. Gain manipulation of visual feedback

Here, we describe the primary experiments that have been reported to date using the Dome system [10]; these experiments and their associated data form the basis for the additional validation analyses we perform in this paper.

The Dome was originally developed to test whether the path integration gain is a plastic variable that can be recalibrated by feedback from landmarks [10]. To achieve this, the relationship between the movement of the animal with respect to the laboratory and the corresponding movement of the landmarks was modulated experimentally. This relationship, termed the experiment gain, was defined as:

(1) $$G_{\text{exp}}=\frac{\text{Displacement of rat in landmark frame}}{\text{Displacement of rat in laboratory frame}}$$

**Figure 4:**
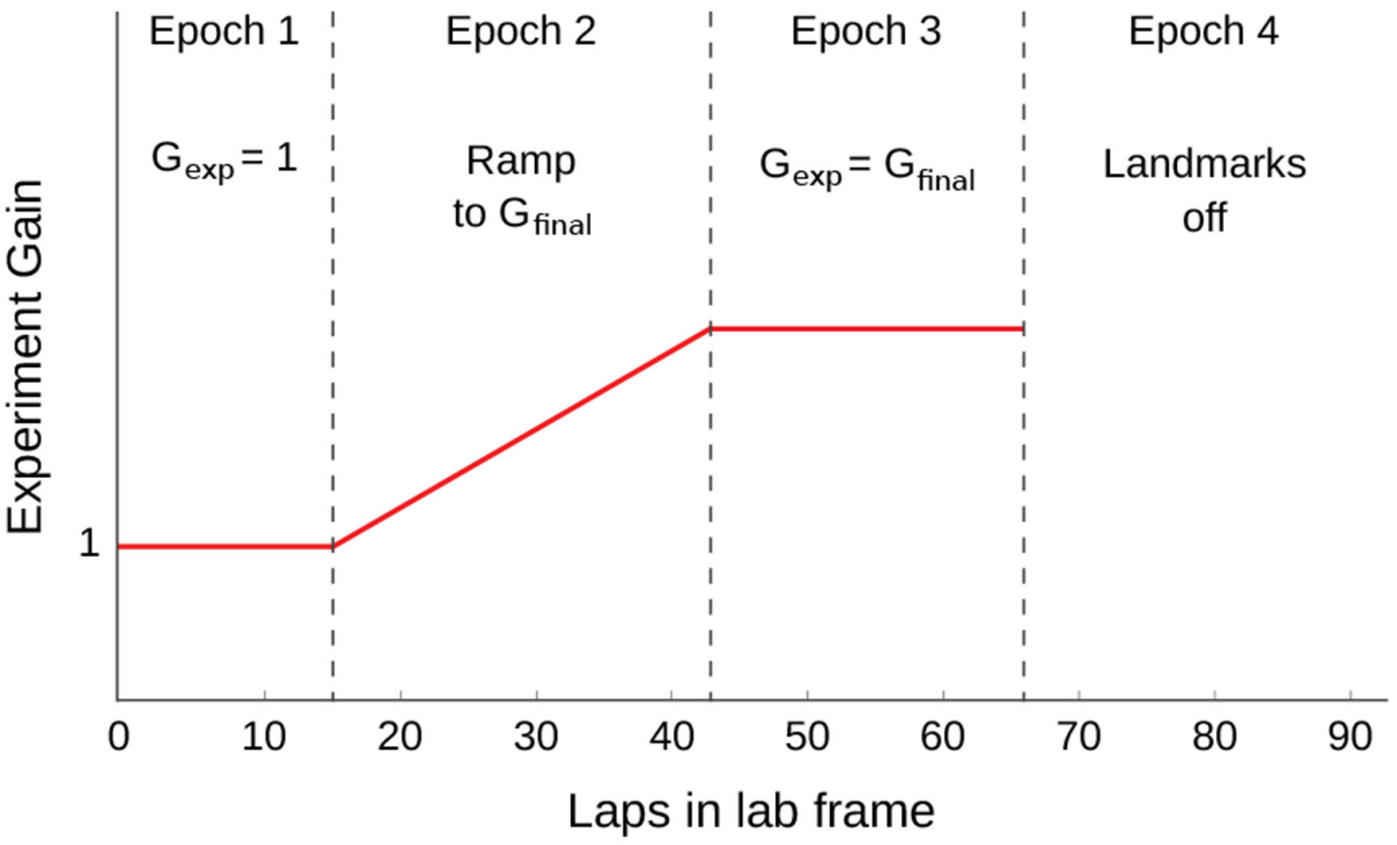
Illustration of the profile of the gain, *G*_exp_, during a typical recalibration experiment [10]. During Epoch 1, the gain was initiated at *G*_exp_ = 1, corresponding to stationary landmarks. In Epoch 2, the landmarks began to move according to an ever increasing or decreasing gain. During Epoch 3 a final experimental gain, *G*_exp_ = *G*_final_, was maintained. Finally, the landmarks were extinguished during Epoch 4. The number of laps for each Epoch is illustrative and varied across actual experiments.

In [10], each experiment session was divided into 4 epochs (Fig. 4). Landmarks were specified to be visible in Epochs 1-3 and turned off in Epoch 4. In Epoch 1, *G*_exp_ was held at 1, meaning that the landmarks remained stationary. During Epoch 2, the experimental gain was ramped up to a constant value over the course of a predefined number of laps. During Epoch 3, the experimental gain remained steady at the final value that it ramped to at the end of Epoch 2. The experiment protocol was defined at the start of the session and execution was fully automated. See supplementary material (Fig. 9) for place cell activity during an example gain manipulation session.

**Figure 5:**
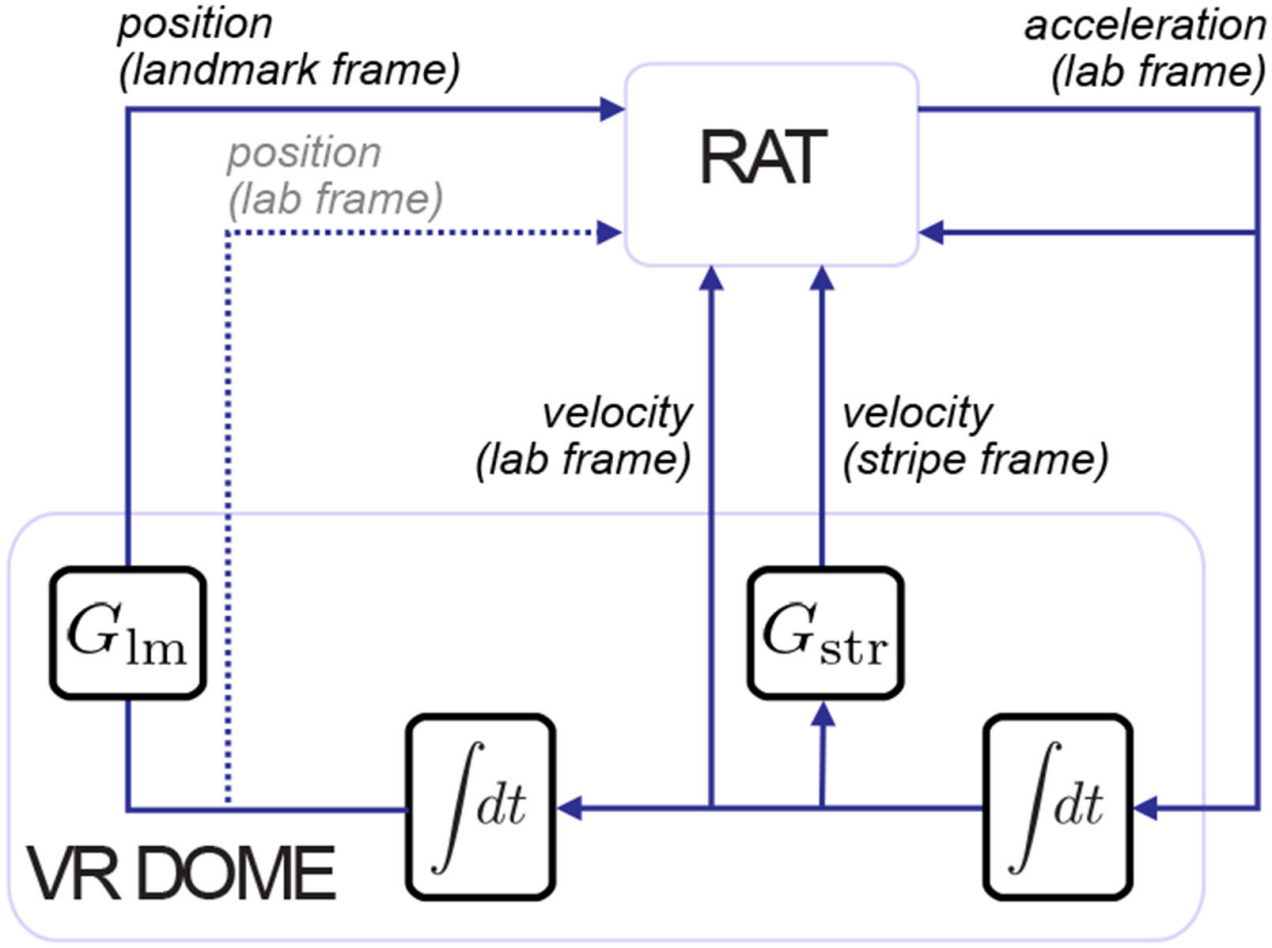
Dome experimental feedback diagram. The blocks represent abstract computational elements and the lines represent how signals flow through the system. From an experimental perspective, we assume that the rat’s output is the force it applies to the tabletop, which is scaled acceleration due to Newton’s laws. Acceleration integrates to velocity which integrates to position. Typically, the rat receives feedback in the form of position, velocity, and acceleration cues in the absolute (laboratory) frame. Our movement of visual cues sets up two additional frames, relative to landmarks and stripes respectively. These frames are determined by their respective (experimentally applied) gains, *G*_lm_ and *G*_str_. Stripe frame information is available to the rat only as optic flow velocity. Information in the landmark frame is available through relative visual position. Through the design of the apparatus, we have weakened information about position in the lab frame (dashed line).

One also can also use the Dome to present optic flow cues. In unreported experiments, we present a set of 80 equally spaced stripes in lieu of polarizing visual landmarks. Since each stripe is indistinguishable from its neighbors, they provide velocity information through optic flow but do not provide position information. The gain of the stripe cue can be manipulated in the same way as landmarks, as a ratio between its displacement and the rat’s displacement. This affords independent experimenter control over two variables: landmark gain (*G_lm_*) and stripe gain (*G_str_*). Fig 5 shows the abstraction of the flow of information within the dome in this condition—the rat is still presumed to have little-to-no direct access to the true position in the lab frame; however, it has direct access to (virtual) position as informed by projected visual land-marks, velocity as informed by stripes, and (true) lab velocity as informed by non-visual path integration cues such as vestibular, proprioceptive, and motor efference copy. We will report the full nature of scientific results from these manipulations in a future manuscript; however the behavior of the animal under stripe manipulation and in the *Current* configuration of the Dome is discussed in Animal Behavior.

### 2.11. Harnessed configuration in Legacy version

In the *Current* version of the Dome, the animal is unharnessed. In the *Legacy* version, the rat was attached physically to the back boom arm. Two ‘chariot arms’ were attached to the boom arm. The rat was trained to wear a body harness, and the harness was fixed to these arms using velcro straps. This partially body-restricted setup allowed the rat the freedom to move forwards, stand up, groom, and turn its head. In addition, the rat could move radially and turn its body by a small amount due to the flexibility of the chariot arms and harness. The inertial load on the rat included the weight of the carbon fiber boom arm and the plastic chariot arms. The boom arm was rotated on a low-friction central bearing, and its angle relative to the central pillar was monitored by an optical encoder. The central pillar was mounted on a smooth bearing as well, and a motor was controlled to keep the relative angle between the boom and pillar near zero. The angle by which the central pillar was rotated was monitored by a second optical encoder. The angle of the rat relative to the laboratory frame was thus the sum of the angle of the central pillar relative to the lab (absolute angle) and the angle of the boom arm relative to the central pillar (relative angle). This system, relying on a pair of optical encoders, allowed the rat’s position to be read accurately and reliably at a high temporal rate without needing real-time optical tracking solutions.

Eliminating the harness in the *Current* configuration of the Dome substantially improves animal behavior and simplifies experiments and training. In the *Current* dome, the animals no longer need to be acclimated to a harness and attached to the chariot arms while running. The behavior is therefore easier to train and easier to maintain over a longer period. Although we found it easy to train rats to accept a harness, it was also typical for the rats to develop stereotyped movements during training and experimental sessions that caused constrictions and abrasions. This sometimes resulted in abrupt and irrecoverable behavioral decline. Freeing the animals of the harness and chariot system eliminated these behavioral inconsistencies. Moreover, the animals are afforded an unrestricted range of natural head and body movements, which is in line with the overall design philosophy of the Dome.

## 3. Experimental Validation

While the specific characteristics that make a VR system “valid” for scientific research is not a well posed question, our goal here was simply to demonstrate the general qualitative normality of neural activity and behavior within the dome, and characterize how neural and behavioral variables change as a function of the experimental gain in Eq. (1). We also sought to ensure that the engineering artifacts were minimized to the extent feasible. Toward these ends, there were three general lines of validation that we considered in this paper. The first two relate to the biology; specifically, we examined how behavioral and neural activity were affected by the VR Dome. The third aspect relates to the engineering: we characterized the latency in visual feedback introduced by the Dome.

### 3.1. Animal Behavior

The running behavior inside the *Legacy* Dome was characterized in [10] (72 sessions, *N* = 5 rats) and reproduced in Table 1. In these sessions, the ramp rate of the experimental gain (*G*_exp_) in Epoch 2 was constant for each rat. Thus, the length of each session was dependent on the amount of deviation of the final *G*_exp_ from its initial value of 1. Consequently, the distance traversed in physical space during a session was highly variable (median = 251.7 m, min = 53.1 m, max = 624.9 m).

In the experiments using the *Current* version of the dome, the only restriction on the animal was a spatial constraint to run on a circular track. There were no restrictions on body or head movement, other than potential twisting of the wired recording tether. The animal was able to orient in any direction that it chose and freely perform behaviors such as grooming, head scanning, and standing up on its hind legs. A video of the rat running in the apparatus is provided in supplementary video 1 (caption: Fig. 8). Using this setup, we collected a currently unreported dataset where the rats were subjected to gain manipulation as in Eq. (1) under moving optic flow cues (38 sessions, *N* = 5 rats). The aggregate behavioral data from this dataset is also reported in Table 1. The median distance traversed by the rats in physical space across all sessions was 388 m (min = 313 m, max = 462 m). As can be seen from the table, the unrestricted behavioral parameters from the unrestricted dataset from the *Current* configuration (last row) are similar to the harnessed (*Legacy*) configuration (first four rows).

After hyperdrive implantation surgery and subsequent recovery, the rats underwent almost daily tetrode turning and training sessions. Training was done in order to achieve a pre-determined behavioral criterion (40 laps run without intervention). We lack complete records for the training sessions; however, knowing that turning and training occurred in tandem, we counted the number of turning sessions as 19.2 ± 5.9 days for the *Legacy* configuration and 12.0 ± 3.5 days for the *Current* configuration. This indicates that training time in the *Current* dome is significantly shorter (*p* = 0.024, *N* = 5, Wilcoxon rank-sum test).

### 3.2. Characterization of place fields under visual manipulation

In [10] it was reported that in the presence of a rotating set of landmarks, the spatial information score [28] of place cells for each animal in the dataset was higher when calculated relative to the landmark frame of reference than the lab frame of reference, a quantitative verification that the place cells most often continued to fire in consistent locations relative to the landmarks. As previously reported, all simultaneously recorded place cells behaved as a single coherent population in all 72 sessions and this ensemble stayed locked to the landmark frame in 60 of 72 sessions across a range of experimental gains, *G*_exp_. It was also reported that the spatial tuning of interneurons was also locked to the visual cues and had the same gain as the simultaneously recorded place cells.

Here, we further characterize the effect of gain manipulation on place fields, in both the lab and landmark frames of reference. We examined place field size, peak firing rate, and spatial information score in the two frames of reference. In addition, we investigated the effect of gain manipulation on field remapping, spatial drift, gross interspike interval distributions, and theta phase precession. The dataset for these new analyses was from the sessions reported in [10] under landmark control, as defined previously.

**Figure 6:**
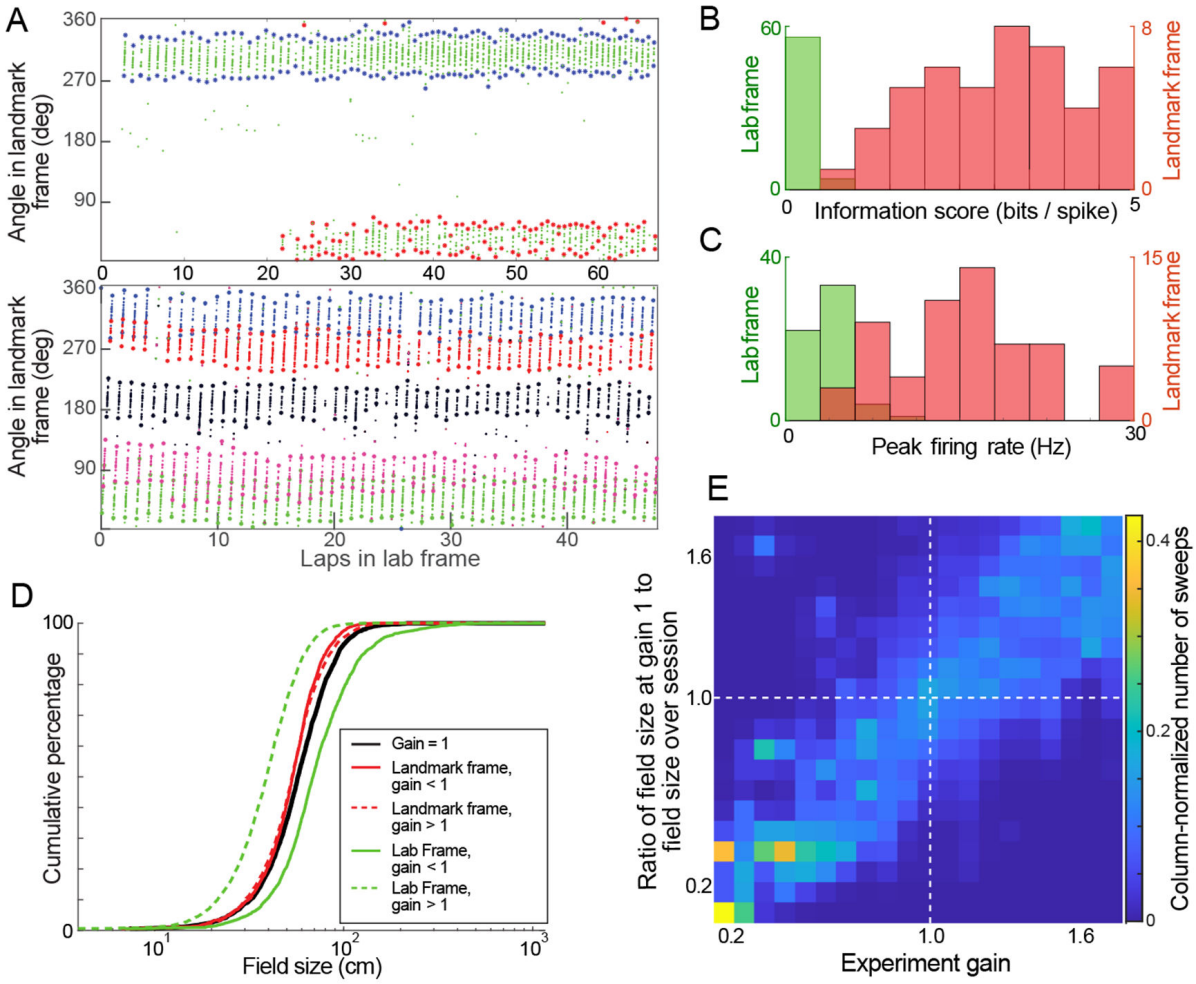
Characterization of place fields in the Dome. (A) Example of automated detection of sweeps through place fields and segmentation into different fields. Plots shows spikes from place cells (small dots) in two sessions plotted as a function of total laps run during a session (x-axis) and angle relative to the moving landmarks (y-axis). Asterisks show the extent of the automatically detected sweeps through the fields. The top plot shows a control session control session(*G*_exp_ = 1), with spikes from one place cell (green dots) with two fields, one which reliably fired in every lap (blue), while the other was potentiated partway through the session (red). The bottom plot shows a gain manipulation session with 5 place cells (5 colors) whose fields cover the landmark frame of reference. This is the same session shown in Fig. 9. (B) Histograms of spatial information score of place fields with high firing reliability computed in lab (green) and landmark (red) frames. (C) Histograms of peak firing rate of place fields with high firing reliability computed in lab(green) and landmark(red) frames. (D) Cumulative distribution of sweep sizes. The black curve represents the distribution of sweep(individual pass through firing field) sizes when *G*_exp_ = 1(2977 sweeps). Green curves represent the field sizes in the lab frame when *G*_exp_ < 1(solid) and *G*_exp_ > 1 (dashed). Red curves represent the sizes of the same sweeps in the landmark frame of reference. (number of sweeps: *G*_exp_ > 1 : 4710; *G*_exp_ < 1: 2071). The distribution of sweep sizes in the landmark frame stayed more closely aligned with those in the control condition(E) Scaling of place fields. From place cells with one field that fired for the duration of Epochs 1-3, we plotted the 2D histogram of the place field scaling factor against the experiment gain of each sweep(n=4535 sweeps). To account for a heterogeneous distribution of sweeps across the gain range, the column bins are normalized by the sum of sweeps binned in the column. The data is distributed around the unity line, demonstrating that place field size scaled with the experimental gain.

We developed a custom algorithm to identify boundaries of individual sweeps, or individual passes, through a place field (Fig. 6A; a preliminary version of the algorithm was reported in [29]). Specifically, a firing rate analogue was constructed by convolving the spike locations in the unwrapped landmark frame of reference by a kernel density function (MATLAB’s ksdensity function, Gaussian kernel sampled at 0.5° bins, bandwidth = 8). The peaks in this curve were detected. Sweeps were defined as contiguous angular bins around each peak where the firing rate analogue was above 10% of the maximum. The sweeps were constrained to be between 5° and 120° in the landmark frame. These limits were not to say that smaller or larger place field sweeps do not exist, but rather were chosen as thresholds to compare between spatial tuning in lab and landmark frames of reference. The next step was to cluster the sweeps as belonging to place fields. This allowed for identification of multiple fields of a place cell, tracking drift of a place field, and localization of remapping events.

For each sweep, the center was defined as its geometric midpoint, which was not necessarily where its peak firing rate occurred. The algorithm iterated through the detected sweeps and, for a given sweep, identified sweeps within a 15 lap window centered on the selected sweep (excluding the lap of the selected sweep). All sweeps within this window were defined to be in proximity of the selected sweep if their geometric center was within 15^°^ of the selected sweep in wrapped angular coordinates in landmark frame. If the number of sweeps in proximity was less than 3, or if the number of spikes in the sweep was less than 4, the selected sweep was deleted as it may be due to spurious spikes that did not maintain stable spatial tuning for a significant period. Sweeps were thus clustered into sets such that any given sweep in the set was in proximity to at least two other sweeps in the set. Each such set was denoted as a place field; in this way, each sweep was considered as one traversal of its associated place field.

This sweep-detection and field-construction algorithm was used to identify the fields (and their sweeps) in the landmark frame for the duration of Epochs 1-3 in the datasets [10] where the fields stayed locked to the landmark frame. The field widths and location for each of these passes were quantified in landmark and lab frames. This allowed quantification of the scaling of place fields in the lab frame of reference, reliability of firing, and spatial drift. As a final verification step, the detected fields were manually curated and merged when it was deemed by visual inspection that an algorithm split one place field into multiple fields. Such manual curation was only needed for about 6% of the place fields, and is similar to the curation process commonly employed after automated spike sorting.

A reliability score of firing for a place field over a given duration was defined as

$$r_{p}=\frac{\#\;\text{of laps in landmark frame place field was active}}{\#\;\text{of laps rat ran in landmark frame}}.$$

The reliability score was used as an indication of stable versus remapping fields. For example, a reliability score of 0.5 indicated that the field was present for half the duration of Epochs 1-3. Note that this measure is not entirely indicative of temporal stability, as the temporal duration of laps in the landmark frame depended on the rat’s velocity and on the experimental gain of the particular session due to the constrained ramp rate of gain in Epoch 2.

Fig. 6B and C compare spatial information scores and peak firing rates in landmark and lab frames. Place cells with a single place field that fired with a high reliability (*r_p_* > 0.9) were selected (*n* = 60). As both frames of reference are coincident in Epoch 1 at *G*_exp_ = 1, spikes from this epoch were discarded.

Spatial information scores for each unit were computed from its firing rate in lab and landmark frames. The information score [28] was defined as

$$\frac{1}{B}\sum_{i=0}^{B}\lambda_{i}\;\log_{2}\;\frac{\lambda_{i}}{\lambda }$$

where *B* was the total number of bins (*B* = 72 for this analysis), *λ_i_* was the occupancy corrected firing rate in bin *i*, and *λ* was the mean firing rate for the unit. Spatial information was greater in landmark frame than in lab frame (Fig. 6B , Landmark frame: median = 3.59, s.d = 1.65, Lab frame: median = 0.22, s.d = 0.14), as expected based on the place field locations being locked to the landmarks when *G*_exp_ ≠ 1 in Epochs 2 and 3 (visual landmarks moving relative to lab).

Firing rates (spikes/s) were computed for each unit using all the data where the rat’s speed exceeded 5 cm/s. The track was divided into 72 bins (5° width). The firing rate during a session for a unit was the number of spikes within each bin divided by the time that the rat spent in that bin. Peak firing rates of the units was greater in landmark frame than in lab frame (Fig. 6C, Landmark frame: median = 15.39, s.d. = 6.27, Lab frame: median = 3.6, s.d. = 1.68)

The cumulative distribution of sizes of sweeps through firing fields in the control condition (Epoch 1, *G*_exp_ = 1) and in the gain manipulation conditions (Epoch 2,3, *G*_exp_ ≠ 1) showed that the distributions of sweep sizes more closely resembled the control conditions when computed in the landmark frame of reference as compared to the laboratory frame (Fig. 6D).

From place cells that fired with a single place field for the entirety of Epochs 1-3 (subset of place cells used in Extended Figure 5 of [10]), we computed a measure of place field scaling for each sweep:

$$f_{p}=\frac{\text{average field size in Epoch}\;1}{\text{size of individual sweep}}.$$

We assigned a gain, *g_p_* to each sweep as the average of the experiment gain, *G*_exp_, at the start and the end of the sweep. If the size of the place field increased in proportion to *G*_exp_, we expected that the *f_p_* for a sweep would be the same as its *g_p_*. The plot of *f_p_* against *g_p_* for *n* = 4535 sweeps (Fig. 6E) showed that this was indeed the case—the field size in the lab frame scaled in correspondence with the applied experiment gain.

**Figure 7:**
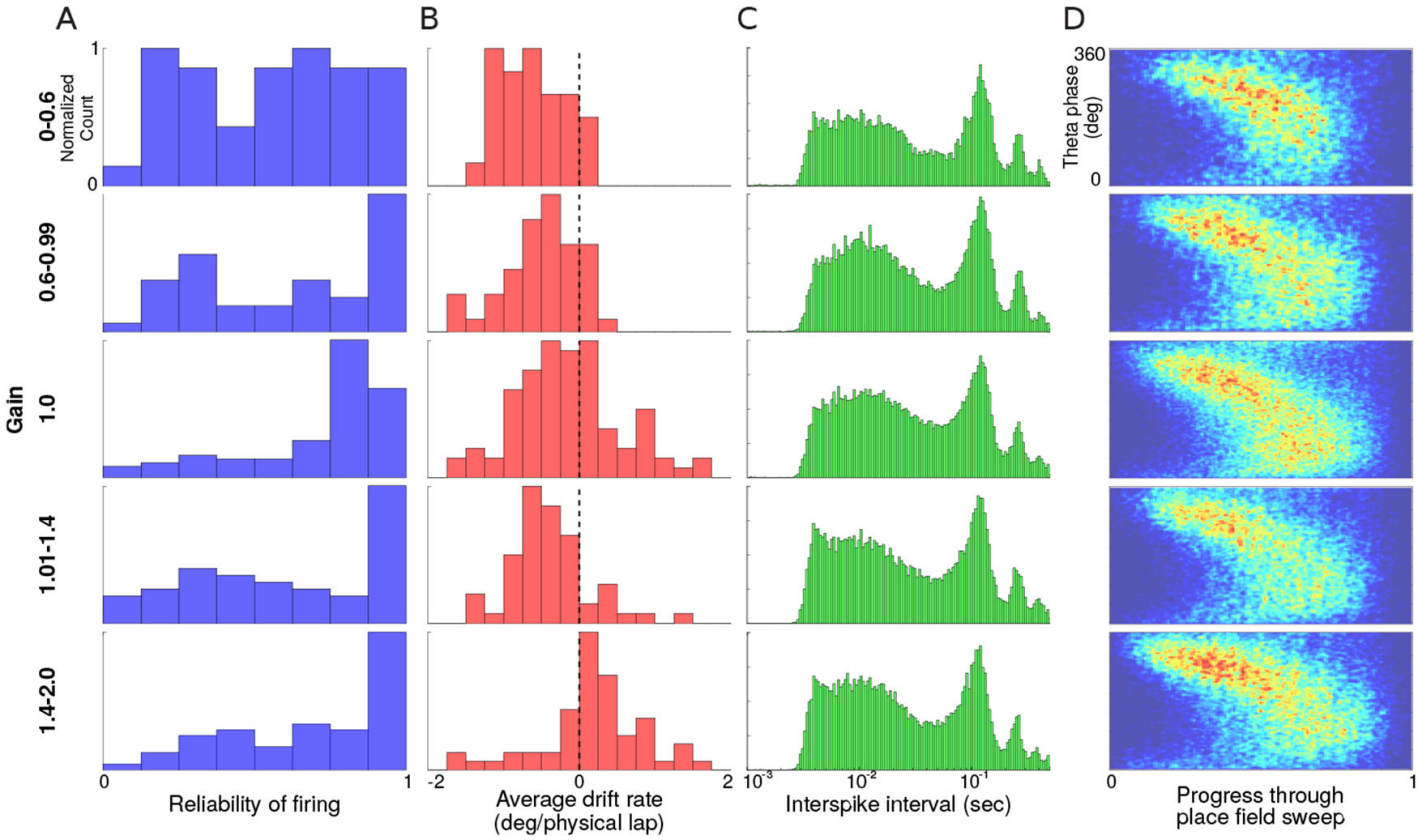
Place field sweep metrics. The rows show data from different gain ranges. (A) Histogram of reliability scores, *r_p_* for place fields (top to bottom, *n* = 42, 48, 94, 59, 60) (B) Histogram of spatial drift rate of the same fields in landmark frame. (C) Distribution of inter-spike intervals for all spikes in all sweeps for all place fields (top to bottom, number of spikes *n* = 12224, 36047, 73111, 36142, 33663). Plot shows a strong peak in the theta range irrespective of gain. (D) Average theta precession. 2D histogram of theta phase of spiking. The x-axis represents normalized extent of a sweep, and y-axis represents theta phase. The theta phases of all spikes from sweeps of place fields are binned in these figures (top to bottom, number of spikes *n* = 13875, 23831, 68312, 35686, 36909). Theta phase precession during sweeps appears unchanged at different gain ranges at this level of analysis.

**Figure 8:**
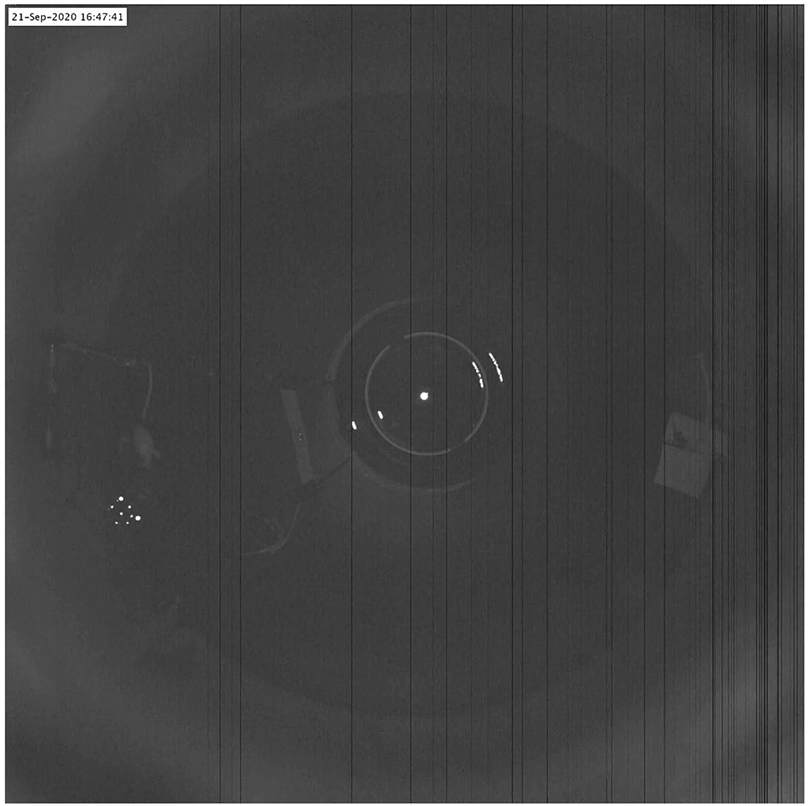
(Supplementary Video 1): Rat running inside the dome apparatus. Video was captured using an overhead camera. A 30s. clip was contrast-enhanced and a timestamp was added (top left corner). Contrast enhancement was necessary since the video is captured at low exposure to prevent saturation of the tracking markers attached to the head of the rat. The large dark circle is the table top and the circle with the bright border at the center is the hemispherical mirror. The rat runs on the outer periphery of the table in the space enclosed between two radial boom arms. The third boom arm, with a paper towel attached for cleaning the table can be seen on the opposite side of the rat. As the rat runs, this camera image is used to track the position and orientation of the crown of markers and the position is used to actuate the central pillar. This is a wired recording configuration: the recording tether is routed through the back boom arm and above the rat before being attached to the hyperdrive implant. Liquid reward is dispensed at pseudo-random intervals and the rat can be seen periodically stopping to lick up the food before continuing to run.

**Figure 9:**
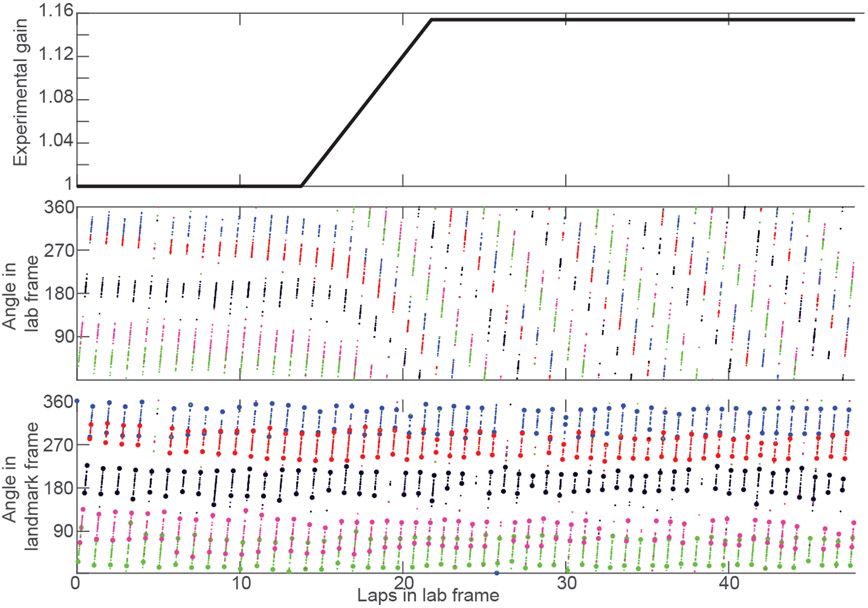
Example gain manipulation session. The x axis for all plots denotes the laps that the rat ran in the laboratory frame. The top plot shows the experimental gain (black), which was 1 for the first epoch, ramped to *G_final_* = 1.153 in the second epoch and maintained this value in the third epoch. The same spikes from 5 units (colors correspond to units) are plotted with respect to their angle in the lab frame (middle plot) and angle in the landmark frame (bottom plot). In the landmark frame, the bounds of automatically detected sweeps are denoted by the correspondingly colored asterisks. Although the firing fields of these neurons drift in the laboratory frame as soon as the experimental gain moves away from its initial value of 1, the fields occur at the same locations in the landmark frame, i.e. they are locked to the landmark frame and exhibit landmark control. The bottom plot is identical to one of the plots in Fig. 6A

We binned place fields into gain ranges based on the *g_p_* of their last sweep. This assigned the place cell to the last gain at which it was active—a conservative measure since during the sessions, the gain always ramped away from its initial value of 1. A histogram of reliability scores *r_p_* for place fields in different ranges of gain (Fig. 7A) showed that at experimental gains away from 1, place fields became less reliable, with a more pronounced effect at the lowest gain bin of 0 — 0.6, similar to what was reported in [30]. Note that this did not mean a loss of spatial information in the landmark frame, which remained high, as shown before in Fig. 6B. Instead, this indicated a higher amount of remapping during more extreme gain manipulations and any newly potentiated fields remained locked to the landmark frame.

We had previously reported that in the presence of visual landmarks, although the place fields were locked to the landmark frame of reference, there was a cumulative drift of the place fields relative to the landmarks that was correlated with final experimental gain (see Extended Data Figure 5 of [10]). This drift was likely indicative of a ‘tension’ between the conflicting position estimates from path integration inputs and the more dominant visual landmarks. To examine this further, we computed the drift rate of place fields in the landmark frame as

$$d_{p}=\frac{\text{ang}(m_{\text{end}},m_{\text{start}})}{\#\;\text{of laps in lab frame}},$$

where *m_end_* was the mean position of the last three sweeps of a place field in the landmark frame, *m_start_* was the mean position of the first three sweeps, and *ang* was the angular distance between these values. In Fig. 7B, the gain bin was again determined by the gain at which the last sweep of the place field occurred. This provided an indication of the general trend of the drift in different gain regimes and was not a fine-grained analysis of the drift as a function of gain and path distance. The drift rate moved from negative values at low gains to slightly positive values at higher gains. This result further supported the ‘tension’ hypothesis—the place fields drifted in the direction expected to relieve conflict between visual landmarks and path integration cues. Fig. 7C shows the histogram of interspike intervals of spikes of place cells at different ranges of gain when landmarks were visible (excluding interspike intervals greater than 0.5s). Peaks were seen corresponding to theta frequency (and its harmonics). Since theta frequency coupling was evident from spiking activity, we computed the theta phase of firing for each spike. During recordings, one channel from each tetrode was sampled at 30 KHz. This signal was down sampled to 250 Hz and filtered to the theta band (MATLAB filtfilt command, butterworth filter, 15*^th^* order, passband 6–12 Hz, stopband width 3 Hz). Theta phase was extracted as the angle of the analytic signal (computed using MATLAB hilbert command), and the phase was shifted so that the mean phase at the peaks of the filtered signal was 0. For spikes from all units recorded on a tetrode, the theta phase of firing was computed by interpolating theta phase to the spike time. Fig. 7D shows the theta phase of spikes between Epochs 1–3 in sweeps from all place cells. By normalizing the x-axis to the extents of each sweep, one observes that the general structure of phase precession of firing is preserved—the theta phase of firing decreased as the animal ran through a place field. Theta precession appeared to occur with the same overall structure at all gain ranges. These analyses demonstrating the overall conservation of theta-phase precession do not preclude possible idiosyncratic modulation of theta-phase related timing of place cell spiking activity by the experimental gain condition manipulation; this remains an area of active investigation.

### 3.3. Visual latency

In the *Legacy* version, the latency between movement of the animal to corresponding movement of the visual scene was measured by actuating a robot tied to the boom arm and measuring the movement of the robot and the corresponding movement of the visual cues from the same set of camera images. The latency was found to be ~97 ms [10].

In the *Current* version, the three-dimensional head position of the rat was tracked in real time by a custom single-camera-based tracking algorithm. The tracking accuracy of the algorithm was validated with a industry-validated visual tracking system and reported in [24] (a full report of this tracking system is in preparation). Traditionally, camera-based tracking systems are slower than tracking systems based on sensors such as optical encoders. The frame rate of the camera as well as the speed of the image-processing pipeline are typical bottlenecks. The Dome uses a camera capable of providing full frame rate (2048×2048) images at 90 frames per second, and our custom tracking algorithm kept pace with the camera up to 81 frames per second (~ 12 ms image processing pipeline). The camera was typically operated at 45 frames per second—this struck a good balance between data size, tracking reliability, and accuracy.

The latency between the movement of the rat’s head and the corresponding movement of the visual cues was measured by manually moving the crown of tracking markers; this crown was typically mounted on the recording implant on the rat’s head. Our software tracked the crown and moved a projected visual landmark in response to the crown position at a gain of 3 (the visual cue moved three times as fast as the crown in the opposite direction). The movement of the crown and the corresponding movement of the visual landmark were both captured by a camera placed near the track. By capturing both the input (crown) and output (cue) movement in the same set of camera frames, we were able to accurately estimate the latency from the movement of the crown to movement of the visual scene. Latency was quantified as the peak of the cross-correlation of the movement of these two objects. Trials were performed at various frame rates of the camera (30, 45, 60, 75 and 90 frames per second). Latencies were found to be between 100–110 ms, marginally slower than our previous encoder-based tracking method. Crucially, the frame rate of tracking had a negligible effect on total latency of visual feedback. This provides flexibility in selecting a frame rate appropriate for examining the behavioral parameters of interest without concerns of significantly altering tracking latency.

To put this latency in perspective, it is useful to consider the amount of spatial error that it can introduce. Note that the amount of error depends on three factors: the latency, the animal’s running speed, and the experimental gain. The angular speed that the landmarks rotate on the surface of the dome is given by the animal’s angular running speed times ∣1 − *G*_exp_∣; if we consider an extreme experimental gain manipulation of either *G*_exp_ = 2 or *G*_exp_ = 0, then the landmarks move at the same angular rate as the animal, in either the opposite or same direction, respectively. Typically, the animals run at about 25 cm/s (Table 1), which corresponds to about 20 deg/s as they circumnavigate the table. If that motion is delayed by 100 ms, it corresponds to an error of about 2°. Given that the dome is 2.3 m in diameter, in these circumstances the latency introduces a spatial error in the projection of the visual scene of about 4 cm at the equator of the dome.

## 4. Discussion

The Dome is a virtual reality apparatus that enables flexible investigation of sensory contribution to the cognitive map in the rodent hippocampal formation. Unlike contemporary rodent VR apparatuses, the Dome is designed to have rats physically locomote, thus maintaining intact path integration inputs. Every physical element of the Dome is either circularly symmetric with respect to the vertical (*z*) axis or is rotating around that axis along with the locomoting rat, so as to minimize the information the animal has about its angular position on the track, except as desired (Design overview and subsystems). We strive to maintain this illusion in multiple sensory modalities, including visual, olfactory, auditory, and tactile. This ensures that the only polarizing sensory cues that the rat receives—i.e. the only information it has about its angular position on the circular track—is from projected visual cues and its own perception of non-visual self motion. This apparatus, while critical to investigating the relationship between path integration and visual landmarks, brings with it a set of challenges in experimental design, our solutions to which are laid out in this manuscript. The software and control design of the Dome allows it to be readily modified to add manipulations of further sensory modalities, and recording of more complex behavioral data (Software and Control).

### 4.1. Applications of the Dome to investigate neural correlates of path integration under naturalistic movement conditions

Using the Dome, we previously showed that the path integration gain, as measured from the activity of a population of hippocampal place cells, is a highly plastic variable that can be recalibrated by landmark cues [10]. It was shown previously that neural correlates in the hippocampus maintained their spatial tuning fields in environments with impoverished external landmarks or even in the dark [31, 32, 33]. This suggests that rats utilize self-motion inputs such as optic flow, vestibular inputs, proprioception, and motor efference, in order to update the neural representation of spatial position [34]. These cues provide information about motion and not position and must be integrated over time to update the representation of spatial position [35, 36]. In addition, the integrated signal has to be calibrated with respect to motion in the real world. This calibration can be achieved through tuning a ‘path integration gain’ factor—the ratio between movement of the animal through the world to the corresponding movement of position in its internal representation [10, 30, 37, 9, 38, 39].

Using the Dome, we can present visual cues in the form of a set of polarizing visual landmarks and a set of stripes that define a purely optic flow cue. The gain of each set of visual cues can be manipulated independently. As shown in Fig. 5, these gains likely modulate distinct streams of spatial information; by manipulating them independently or in concert, we can quantify the pair-wise interactions between visual landmarks, optic flow, and the remaining path integration cues. Such manipulations would allow us to introduce conflict between (virtual) visual cues and (naturalistic) path integration cues, and quantify their relative contributions as well as influence on each other in informing the cognitive map.

In short, we find that the animals continue to run within the Dome in a naturalistic way, without dramatically changing their behavioral characteristics as a function of the experimental gain. We also find that place cell activity maintains a number of key characteristics reported widely in the literature. While there were modulations of both behavior and neural activity during gain manipulations, the system seems to have been “stretched” and not “torn”: the modulations are certainly interesting and deserve deeper inquiry; however they are also similar enough to known phenomena so as to engender confidence that the neural computations within the Dome are naturalistic.

### 4.2. Comparison with head- or body-fixed VR systems

Traditional VR apparatuses where head- or body-fixed rats run on air-supported balls can simulate essentially infinite two-dimensional visual environments. In the Dome, however, the movement of rats are restricted to a one-dimensional, circular trajectory. As an advantage, rats in the Dome have access to a richer suite of path integration cues. There is usually a trade-off in terms of constraints on behavior versus constraints on the measurements available as a result of the behavior or as the result of the experimental recording techniques afforded by head-fixed or head-free conditions. We deem naturalistic locomotor performance to be crucial investigating the role of path integration in the formation and maintenance of the cognitive map. Consider a head-fixed animal exploring a 2D VR world. The vestibular signal is clamped at a constant value. However, as the animal explores a 2D virtual world, visual inputs signal that the head direction is changing and the head position is moving, creating a persistent conflict with vestibular signals. A similar conflict arises in body-fixed preparations, in which animals can generate vestibular signals by moving their heads. However, these signals are in conflict with visual cues when the animals rotate the visual world by moving the trackball with their legs but do not make corresponding head movements consistent with the rotating cues. Adaptation to the continuous multimodal conflicts in these systems may happen in different ways. Even something as straightforward as down-weighting either of the signals can lead to a cascade shift of neural responses of downstream conjunctive representations and computations. The resultant network dynamics will be at a new equilibrium and can manifest a form of cognitive representation that differs from one under ethologically relevant conditions. Such dynamics may explain conflicting results in the literature between some VR systems that show apparently normal place field firing and others that show apparently normal temporal dynamics of spiking but with no consistent spatial relationship to the environment [6, 5, 37]. Thus, while experiments and manipulations in head- or body-fixed systems have yielded important insights about the neural system under investigation, care must be taken that the results are interpreted in the context of these constraints—indeed, one can find similar cautionary tales in other organisms such as flies [40, 41]. In the context of a laboratory preparation to investigate the cognitive map, recording from a rat behaving as close as possible to its ethologically relevant conditions is crucial to interpreting the recordings to reveal the natural network dynamics [23]. We believe that for spatial navigation in mammals, the Dome can provide a critical link between fully tethered VR systems and completely free behavior, helping ease the comparison across these regimes. Since animals are freely locomoting, the Dome apparatus can be applied across species without needing to alter restraints. However, modifying the dome for other animals will involve some design considerations, such as determining the appropriate size and scale of the apparatus, and ensuring that the position tracking and control bandwidth are sufficient to keep the animal within the radial boom arms.

### 4.3. Low latency modulation of visual feedback

The ability of the Dome apparatus to continuously modulate visual feedback with low latency (discussed in Gain manipulation of visual feedback and quantified in Experimental Validation) was, in our opinion, critical to the success of this experiment protocol. In a natural environment, the animal can use stable landmarks for localization. When the animal moves, these landmarks move across the visual field with a delay corresponding to the time for synaptic transmission. With internal compensation of this delay, the animal can check if the movement of the landmarks is within the time window of association with its own self-movement. The low latency visual feedback of the Dome makes it likely for the visual scene modulation to be perceived as causally linked to the animal’s own movement without experiencing the dissonance of the visual scene changing with a perceivable lag. Additionally, the animal may perceive the change in the visual scene as being causally related to its own movement if the change in the visual scene matches its prediction of how much the visual scene should change, given its estimate of self-movement. If the brain performs a comparison between predicted and actual location frequently enough, the ability to gradually and continuously change the experiment gain becomes crucial. This ability ensures that the error between how much the animal predicts the visual scene to have changed versus how much its actually changed remains small. If this error is small enough that trust in the stability of the landmarks isn’t eroded but still remains consistent and larger than noise, it further enables the plastic nature of the path integration gain to become evident.

### 4.4. Possible future experiments using the Dome

The Dome apparatus is very versatile for experiments involving visual cue manipulation. In addition, our ability to track the rat’s head position and orientation in real-time and at high rates can in principle allow two-dimensional movement and projected cues that simulate three-dimensional objects (See [17, 42] for implemented examples in rats, mice, fish and flies). However, the size of the traversable environment is still limited by the size of the table surface or environment boundaries. In comparison to head- and body-fixed preparations, wired recording and reward delivery will require a more complicated approach in a two-dimensional experiment inside the Dome.

The apparatus also allows for modifications that can bring other sensory cues under the control of the experimenter. Some potential modifications are described below. These experiments are conceptual but will hopefully illustrate the range of possible applications.

1. A bank of speakers placed under the table can provide directional audio cues to the animal. These can be used to generate an auditory landscape in addition to / in lieu of visual landmarks.
2. The double tabletop design allows the upper table to be motorized and actuated while the rat is running on it. This manipulation would allow us to change the gain between vestibular and proprioceptive inputs as a continuous variable. For example, in the special case where the table is moved in the opposite direction of the rat at the same speed (gain = 0, similar to a body-fixed VR), linear vestibular inputs will be largely extinguished while proprioceptive and motor efference inputs will be maintained.
3. Instead of the two boom arms ahead of and behind the rat, we can place the rat on a base with wheels. This ‘car’ configuration could be used to move the rat passively. This would maintain vestibular input while extinguishing proprioceptive and motor efference input related to natural movement. We implemented this configuration successfully and recorded place cells in one animal (not shown). A similar setup was used by [38] to investigate CA1 activity under modified path integration input.
4. As also explored in [38], the rat can be provided with a button to ‘drive’ the car voluntarily, in order to engage attention and maintain the hippocampus in a theta-modulated state.
5. Rotating the table as described above is akin to placing the rat on a treadmill. An additional manipulation can be gained if the rat is actually placed on a treadmill which in turn can be rotated around using the radial boom arm. This system would forgo the increased inertia of a moving tabletop and provide a direct manipulation of vestibular and proprioceptive inputs. The treadmill velocity determines a proprioceptive manipulation, whereas the treadmill movement relative to the laboratory would determine a vestibular manipulation. Previous work in non-human primates has shown that in cases where vestibular measurements of self-motion match the sensory expectations (e.g., due to corollary discharge / efference copy), the vestibular signals are attenuated [43]. This inherent cancellation of vestibular cues needs to be taken into account while interpreting data from such a manipulation where the brain integrates active (rat running on treadmill) and passive (treadmill moving on table) vestibular components.

The current dome hardware and software design allows the incorporation of any of these proposed manipulations. Depending on the scientific question at hand, this flexibility of experimental manipulations will prove crucial to our investigation of the integration and function of the cognitive map. Our intent with this manuscript is to document and make public the detailed hardware and software design of the dome, so that other research groups can take advantage of these rich manipulations that the Dome offers.

## Supplementary Material

All Supplementary Material

## Figures and Tables

**Table 1:** Behavioral parameters. For Epochs 1-4 (Fig. 4), the values were reported in the Extended Data from [10]; this data set comprised *N* = 5 rats. In Epoch 1, the experiment gain, *G*_exp_ is 1. In Epoch 2, *G*_exp_ is ramped from 1 to a target final gain. In Epoch 3, *G*_exp_ is held constant at the target gain. In Epoch 4, the landmarks are extinguished. For Optic flow, the values were computed from an unpublished dataset, *N* = 5 distinct rats. Values are shown as mean (s.e.m.).

Dataset	Velocity (cm/s)	Pauses per lap (s)	Pause duration(s)	Interpause interval (s)	Interpause distance (cm)
Epoch 1	24.6 (0.7)	0.9 (0.2)	8.8 (1.0)	55.8 (8.2)	887 (136)
Epoch 2	25.2 (0.9)	1.0 (0.1)	6.5 (0.5)	61.8 (18.0)	1119 (399)
Epoch 3	25.0 (1.0)	1.5 (0.2)	8.8 (1.0)	26.3 (3.5)	461 (79)
Epoch 4	24.2 (1.0)	1.5 (0.3)	9.2 (0.8)	34.9 (9.4)	531 (125)
Optic flow	23.5 (0.7)	0.8 (0.2)	10 (0.7)	41.0 (6.7)	579 (96)

## Data Availability

CAD (Solidworks) parts and assemblies for the Dome are available at github.com/LIMBSlab/madhav2021dome_domecad ROS nodes that operate the Dome are available at github.com/LIMBSlab/madhav2021dome_domeros
