# Supplementary figures and images for: The Dome: a virtual reality apparatus for freely locomoting rodents
Source: J Neurosci Methods. Author manuscript; Available in PMC 2022 Jun 9. (PMC9178503; doi:10.1016/j.jneumeth.2021.109336)

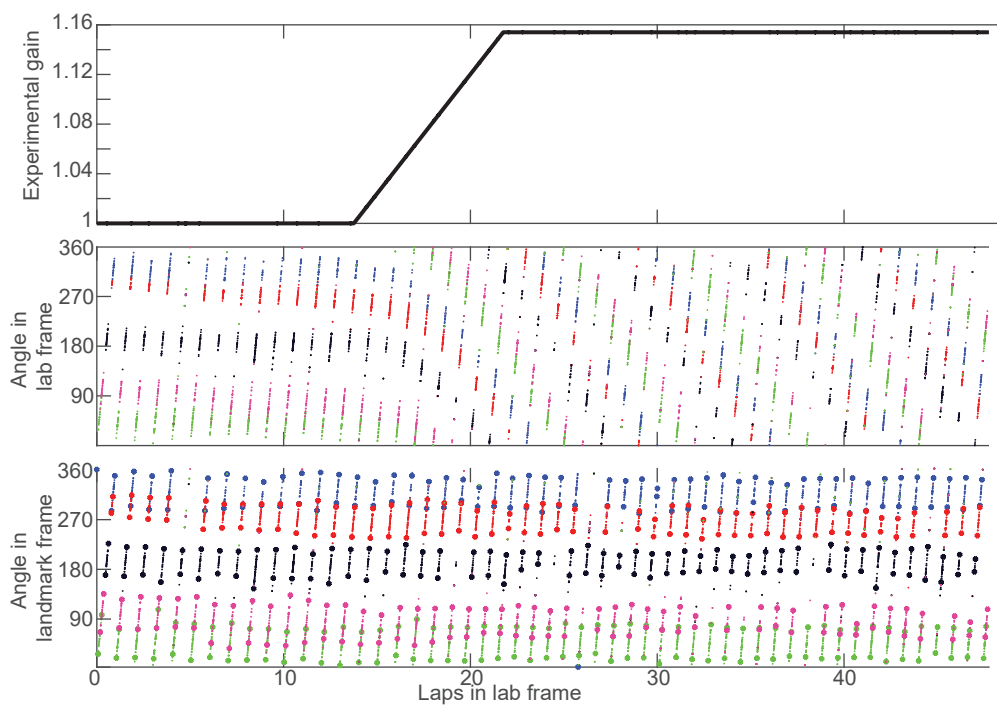

Supplement: All Supplementary Material [file NIHMS1804220-supplement-All_Supplementary_Material.zip › ScienceDirect_files_04May2022_21-12-19/1-s2.0-S0165027021002715-mmc1.pdf]

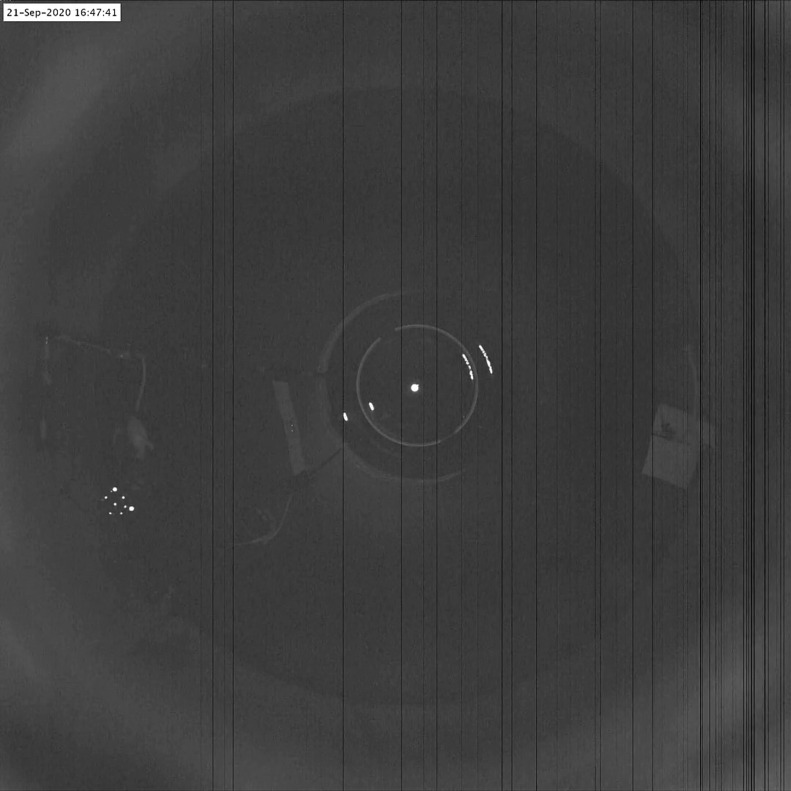

Supplement: All Supplementary Material [file NIHMS1804220-supplement-All_Supplementary_Material.zip › ScienceDirect_files_04May2022_21-12-19/1-s2.0-S0165027021002715-mmc3.jpg]

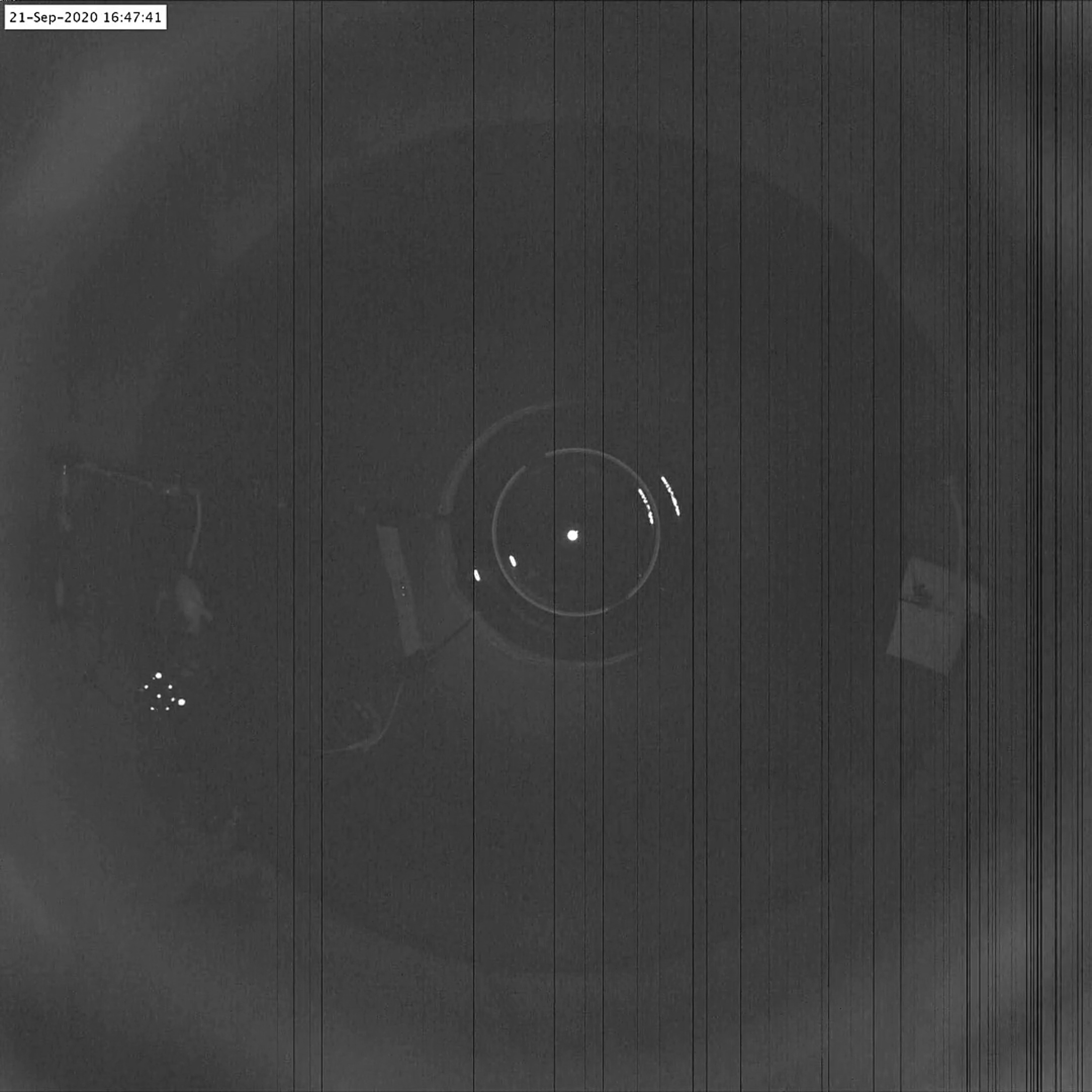

Supplement: All Supplementary Material [file NIHMS1804220-supplement-All_Supplementary_Material.zip › ScienceDirect_files_04May2022_21-12-19/1-s2.0-S0165027021002715-mmc3_lrg.jpg]
